# A Similarity-Based Process for Human Judgment in the Parietal Cortex

**DOI:** 10.3389/fnhum.2018.00481

**Published:** 2018-12-13

**Authors:** Linnea Karlsson Wirebring, Sara Stillesjö, Johan Eriksson, Peter Juslin, Lars Nyberg

**Affiliations:** ^1^Department of Psychology, Umeå University, Umeå, Sweden; ^2^Department of Integrative Medical Biology, Umeå University, Umeå, Sweden; ^3^Umeå Center for Functional Brain Imaging, Umeå University, Umeå, Sweden; ^4^Department of Psychology, Uppsala University, Uppsala, Sweden; ^5^Department of Radiation Sciences, Umeå University, Umeå, Sweden

**Keywords:** judgment and decision-making, fMRI, exemplar model, multiple-cue judgment, cognitive model

## Abstract

One important distinction in psychology is between inferences based on associative memory and inferences based on analysis and rules. Much previous empirical work conceive of associative and analytical processes as two exclusive ways of addressing a judgment task, where only one process is selected and engaged at a time, in an *either-or* fashion. However, related work indicate that the processes are better understood as being in *interplay* and simultaneously engaged. Based on computational modeling and brain imaging of spontaneously adopted judgment strategies together with analyses of brain activity elicited in tasks where participants were explicitly instructed to perform similarity-based associative judgments or rule-based judgments (*n* = 74), we identified brain regions related to the two types of processes. We observed considerable overlap in activity patterns. The *precuneus* was activated for both types of judgments, and its activity predicted how well a similarity-based model fit the judgments. Activity in the superior frontal gyrus predicted the fit of a rule-based judgment model. The results suggest the precuneus as a key node for similarity-based judgments, engaged both when overt responses are guided by similarity-based and rule-based processes. These results are interpreted such that similarity-based processes are engaged in parallel to rule-based-processes, a finding with direct implications for cognitive theories of judgment.

## Introduction

How accurately can we expect humans to combine evidence in order to produce a quantitative estimate of a criterion, as medical doctors, prosecutors, teachers, or brokers are forced to do on a daily basis? Arguably, the answer to that question boils down to how well we are able to describe and predict the cognitive processes and knowledge representations that humans draw on to produce such judgments. Human *multiple-cue judgments* – the estimate of a continuous criterion based on multiple different cues – have repeatedly been shown to involve considerations of abstract rules about how different pieces of evidence relate to the criterion that is to be judged (e.g., Einhorn et al., 1979; Brehmer, 1994; Juslin et al., 2003; Gigerenzer and Gaissmaier, 2011). Such judgment data is well predicted by linear regression models or other so called cue-based, heuristic or rule-based models.

Human multiple-cue judgments have also been shown to involve a different type of strategy, namely the use of associations to memories of previous similar situations and are assumed to be made based on the similarity between the to-be-judged object and similar examples stored in memory. Such judgment data is well predicted by exemplar models (e.g., Medin and Schaffer, 1978; Nosofsky, 1984; Juslin et al., 2003). Categorical judgments (i.e., where the criterion is binary instead of continuous) are traditionally described remarkably well by such models (e.g., Medin and Schaffer, 1978; Nosofsky, 1984; Nosofsky and Johansen, 2000). During the last decade, the field has seen an explosion of studies demonstrating the prevalence of exemplar-based strategies also in multiple-cue judgment (e.g., Juslin et al., 2003, 2008; Olsson et al., 2006; Karlsson et al., 2007, 2008; von Helversen and Rieskamp, 2009; von Helversen et al., 2010, 2013; Mata et al., 2012; Pachur and Olsson, 2012; Hoffmann et al., 2013, 2014). This is usually investigated empirically by pitting detailed cognitive models of the different kinds of processes against each other, using specific task structures to generate different predictions from the models, and classifying participants as more reliant on one or the other type of strategy. These studies have advanced the theory of human judgment by demonstrating the importance of considering not only analytical types of strategies in judgment, but also exemplar-based strategies. However, an assumption inherent in these studies is the conception of similarity-based and rule-based processes as two exclusive ways of addressing a task, where only one type of process is selected and engaged, in an *either-or* fashion. Several authors have even argued for the plausibility of controlled *strategy shifts* (Haider et al., 2005; Rehder and Hoffman, 2005; Meeter et al., 2006; Karlsson et al., 2008).

In contrast, there are empirical observations from the categorization literature demonstrating effects of irrelevant similarity features on accuracy and response times, even when inferences are done following a rule-based strategy (Brooks and Hannah, 2006; Hahn et al., 2010). Results of that kind would appear to contradict an *either-or* view. Indeed, several computational accounts of category learning instead suggest an *interplay* between rule-based and similarity-based processes in categorization. On this view, rule-based and similarity-based processes should be seen as parallel, simultaneously competing to control behavior on a trial-by-trial basis rather than being chosen and executed in an *either-or* fashion (e.g., Palmeri, 1997; Ashby et al., 1998; Erickson and Kruschke, 1998).

Despite an impressive amount of empirical behavioral evidence for the distinction between rule-based and similarity-based processes, both in categorization and judgment, brain-imaging techniques have arguably not been used to its fullest potential to test the validity of the distinction and hypotheses regarding the relationship between the two.

Several of the more recent brain-imaging studies investigating similarity-based processes in categorization have focused on different aspects of this type of process, with the help of model-based approaches. For example, it has been demonstrated that similarity-based processes as captured by an exemplar-based model (*EBM*) might underlie both categorization as well as recognition memory, as parameter changes of the *EBM* could be used to explain the relatively few differences in brain activation between the two types of cognitive tasks (Nosofsky et al., 2012). In the same vein, Davis et al. (2014) managed to demonstrate that an estimate of memory strength based on global neural pattern similarity in medial temporal lobes correlated well with behavioral measures in both categorization and recognition memory. Further, it has been shown that participants’ perception of how typical an exemplar is of its category match very well with a neural typicality measure in occipital and medial temporal lobes, derived by an *EBM* using similarity computation on patterns of brain activation (Davis and Poldrack, 2014). As a last example, Mack et al. (2016) found support for the proposal that representations used for categorization in the hippocampus are dynamically updated depending on the learning context (e.g., when facing a new task goal for which the representations are supposed to be used).

The question regarding a distinction between rule-based and similarity-based processes and hypotheses regarding the relationship between the two, have however not seen the same attention lately. Hitherto, most brain-imaging studies investigating the distinction have tended to focus on discussing the *differences* in brain activity related to the two types of processes (see e.g., Patalano et al., 2001; Grossman et al., 2002; Koenig et al., 2005; von Helversen et al., 2014b). By doing so, it has been suggested that rule-based processes should be associated with relatively higher brain activity in dorsolateral prefrontal cortex, anterior cingulate cortex and/or posterior parietal cortex compared to similarity-based processes, while similarity-based processes should be associated with relatively higher brain activity in anterior prefrontal cortex, inferior parietal cortex and/or posterior cingulate cortex as compared to rule-based processes (see e.g., Patalano et al., 2001; Grossman et al., 2002; Koenig et al., 2005; von Helversen et al., 2014b).

However, focusing only on the relative differences in brain activity between two kinds of processes is misleading because brain activity important for both kinds of processes runs the risk of being canceled out. This might be especially unfortunate when investigating whether a distinction between processes is useful in the first place, and also with regard to how a relationship between them should be construed.

**FIGURE 1:**
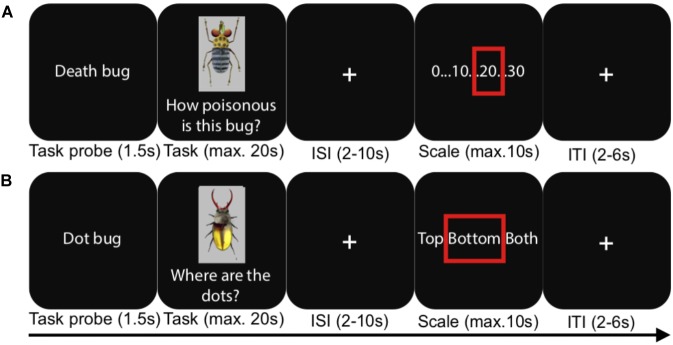
An illustration of the experimental procedure during the fMRI test phases. **(A)** A judgment task trial, **(B)** a visual detection baseline trial. ISI, inter-stimulus interval; ITI, inter-trial interval. In reality, the scale for the judgment task depicted all whole numbers between 0 and 30.

In the present study, we use functional magnetic resonance imaging (fMRI) to acquire neurocognitive data informative for the distinction between rule-based and similarity-based processes in human judgment and how the relationship between them should be construed (i.e., according to an *either-or* view or an *interplay* view). Prior to scanning, participants learned to make *multiple-cue judgments* either using *cue abstraction* (a rule-based process, captured by a *cue-abstraction model, CAM*) or *exemplar memory* (a similarity-based process, captured by an *EBM*, see details on the modeling below) as the basis for judgments. The general task set-up was previously successfully used in numerous behavioral studies (e.g., Juslin et al., 2003, 2008; von Helversen and Rieskamp, 2009) and one brain imaging study (von Helversen et al., 2014b) of multiple-cue judgment, aiming at investigating the reliance on rule-based and similarity-based processes. The task was to judge the toxicity (a pseudo-continuous criterion) of the lethal but fictitious “Death bug” varying on five binary cues (i.e., legs, eyes, back, head and mandibles) shown as illustrations on a computer screen (see Figure 1). The general logic of the task was, as in the previous studies, that the items included in the training and test phases were chosen to yield qualitatively distinct judgment predictions from *CAM* and *EBM*. Specifically, the test phase introduced new items that were not experienced during the training phase. In order to be judged correctly, these items require both intra- and extrapolation (i.e., judgments beyond the criterion range seen in training). *EBM* predicts that judgments on a new test item are based on the similarity of that item to similar items that are experienced during training and stored in memory. Because *CAM*, a linear and additive model weighting and adding the impact of each cue on the criterion, makes no such assumptions, *EBM* and *CAM* predict qualitatively distinct response patterns for the test phase.

In the present study, we had four different conditions in a between-subjects design, taking advantage of the logic of the task set-up just described. As mentioned, prior to scanning, participants learned to make judgments either using a rule-based or a similarity-based process. In two separate experiments, rule-based and similarity-based processes were learnt in one of two different *learning modes*: (*1*) after instruction (“Instructed” conditions) or (*2*) by spontaneous choice by the participants (“Spontaneous” conditions). Strategy adherence was confirmed with cognitive modeling, and the two different learning modes were used in order to capture the core aspects of these processes irrespective of learning mode. After each learning phase, participants in all four conditions performed a test phase during fMRI scanning. The test phase was identical in all conditions and involved judging the toxicity of the Death bugs, without feedback on the correct answers (Figure 1). A visual detection judgment task was used as a baseline and involved detecting whether a dot was apparent on the upper body, lower body or both upper and lower body of a bug.

To address the issue of whether the relationship between rule-based and similarity-based processes should be construed according to an *either-or* view or an *interplay* view, we analyzed the *overlap* in task-related brain activity, i.e., what brain activity that is shared between making judgments with rule-based and similarity-based processes, compared with the baseline task, as identified statistically in conjunction analyses (Nichols et al., 2005; for recent similar approaches in relation to perceptual decision making and category learning see Milton and Pothos, 2011; Mega et al., 2015; Carpenter et al., 2016; Milton et al., 2017). If no overlap exists, or if the overlap does not play a functional role for judgment (i.e., overlapping brain activity is *not* related to whether judgment data is well described by a rule-based model or a similarity-based model), this would support the *either-or* view. On the other hand, if the two types of processes share considerable task-related brain activity and at least parts of this brain activity also play a functional role for judgment (i.e., overlapping brain activity *is* related to model fit), the *interplay* view would be supported. Note that evidence in relation to the *interplay* view would not be possible to obtain if only focusing on the relative differences in brain activity related to the two kinds of processes (e.g., Koenig et al., 2005; von Helversen et al., 2014b).

## Materials and Methods

We conducted two separate experiments using a between-subjects design. Participants learned to make judgments either using a *rule-based process* or a *similarity-based process*, using one of the two learning modes: *instructed* (experiment 1) or *spontaneous* (experiment 2). After learning, in all four conditions, participants performed a test phase in the fMRI scanner. Participants were all right-handed by self-report. The study was carried out in accordance with the recommendations of the Regional ethical review board in Umeå, Sweden. All subjects gave written informed consent in accordance with the Declaration of Helsinki. The study was approved by the Regional ethical review board in Umeå. All data supporting the conclusions of this manuscript will be made available by the authors upon request, without undue reservation, to any qualified researcher.

### Instructed Strategies

#### Participants

Forty neurologically healthy participants (*M*_age_ = 25.8; range = 19 to 34; *SD*_age_ = 4.5; 22 females), with normal or corrected-to-normal vision, were recruited for instruction of rule-based processing (*n* = 20) or similarity-based processing (*n* = 20). Participants received 500 SEK for participation. We discarded data from two participants from the analyses due to extensive head movements.

#### Judgment Task

The task was to judge the toxicity of the “Death bug”. Participants were taught how to do this by either relying on rule-based processing or similarity-based processing.

The Death bugs varied on five binary cues (i.e., legs, eyes, back, head and mandibles) with cue values “-1” or “1” (e.g., the legs can be either short or long). In general, a cue value of “-1” implies that the presence of this cue value decreases the criterion, while a cue value of “1” implies that the presence of this cue value increases the criterion (see Table 1). The criterion values were a linear function of the cues, where each cue is given a different weight. The weights are monotonically decreasing such that some cues more strongly implies a higher or lower criterion value. Thus, in contrast to most categorization studies, in this judgment task it is necessary to attend to more than one cue in order to perform well.

**Table 1:** Items in the instructed strategies conditions (experiment 1).

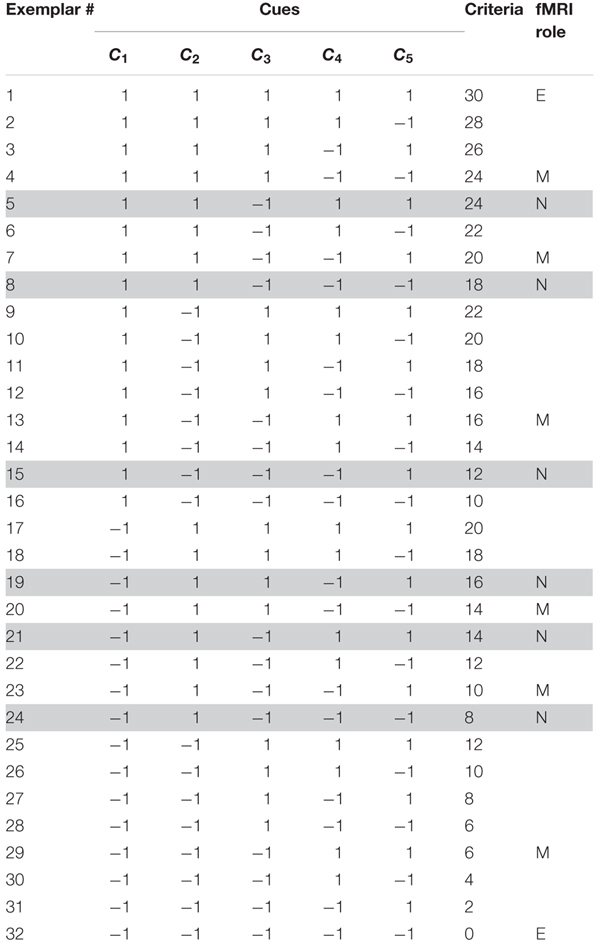

*The table shows the cues and criterion values of all exemplars and the exemplars that were used during the fMRI test. N, new exemplars, included only during the fMRI test for both conditions; M, exemplars included in the memorization phase of the *EBM* condition and during the fMRI test for both conditions; E, extreme exemplars included during the fMRI test for both conditions, but used only for behavioral measures. Marked in gray are items critical for the reported fMRI contrast analyses.*

The criterion values (the toxicity, *c*) in the instructed strategy conditions were given by a linear, additive function of the five cues:

(1) $$c=15+5\times C_{1}+4\times C_{2}+3\times C_{3}+2\times C_{4}+1\times C_{5}.$$

where the importance of each cue is determined by the coefficients 5, 4, 3, 2 and 1, respectively. For example, a Death bug with cue values [-1 -1 -1 -1 -1] will have a toxicity level of 0, while a Death bug with cue values [1 1 1 1 1] will have a toxicity level of 30 (see Table 1).

#### Behavioral Procedure

The procedures for instruction of rule-based processing or similarity-based processing were identical but differed in terms of content. The instructions contained four general phases: (1) a *memorization phase*, where participants either memorized each of the 10 cue-criterion relations, written as verbal statements, or six illustrations of Death bugs together with their toxicity value (exemplars denoted “M” in Table 1); (2) a *recognition phase*, where participants saw the memorized material again and were asked whether it was from the memorization phase or not. Participants had to reach a performance criterion before moving on to the next phase; (3) a *judgment training phase* where participants were taught how to use the knowledge they had just learnt, in order to judge the toxicity of the bugs, and (4) a *repetition phase* where participants were shown the memorized material one last time.

##### Procedure for instruction of the rule-based process

In a *memorization phase* participants were shown each of the 10 cue-criterion relations, written as verbal statements, on a computer screen (e.g., “A striped head strongly implies high toxicity” or “A dotted head strongly implies low toxicity” or “Green mandibles weakly implies high toxicity”). Participants were first shown all statements in a summary table followed by each statement separately on the screen, in random order, self-paced, for a total of five repetitions per statement. Participants were instructed to carefully memorize the importance of each cue value and whether it implied high or low toxicity. In the *recognition phase*, participants saw each statement again, in random order, and were asked whether it was one of the statements from the memorization phase or not, with corrective feedback (right/wrong). This phase contained the 10 original statements and 10 false statements, where the importance and/or the direction of each cue (i.e., whether it implied high or low toxicity) had been altered. The recognition phase continued until the participant had reached 100% correct on a block of 20 statements. In the *judgment training phase*, participants were taught how to use the knowledge of cue-criterion relations they had just learnt, in order to judge the toxicity of the bugs. Participants were instructed that upon viewing a bug they were supposed to consider what they had learnt about all the five cues, starting with the most important cue, in order to come up with a numerical estimate between 0 and 30. Participants were then given a few practice trials (without feedback) for getting acquainted with making toxicity judgments with the newly learnt knowledge. For the first trial, they were told the correspondence between the verbal cue labels and the visual features of the bug. Participants also received a few practice trials to judge bugs with only two cues visible (for a separate manipulation, not reported) and a few practice trials for the cognitive-perceptual baseline task: to judge whether similarly looking bugs had a gray dot painted on the bottom, top or both bottom and top of the body. Finally, in the *repetition phase*, participants were shown all 10 verbal statements again, in a random order on the screen, for 5 s each, in order to make sure they would remember them correctly.

Next, participants were invited to the scanner for a test phase (see below under *Imaging procedure*).

##### Procedure for instruction of the similarity-based process

In a *memorization phase* participants were not shown any verbal statements but instead six illustrations of Death bugs together with their toxicity value (exemplars denoted “M” in Table 1), on a computer screen, in random order, one at a time, for a total of five repetitions per bug. A time limit of 8 s per bug was used in order to minimize the possibility for elaborate cue abstraction processes. Participants were instructed to carefully memorize the illustration of each bug together with its toxicity. Moreover, participants were explicitly told that it was important that they considered the illustration as a whole together with the toxicity and that they should not try to figure out any rules how to relate parts of the illustration to the toxicity value. In the *recognition phase*, participants first saw each bug again without its toxicity value, in random order, and were asked whether it was one of the bugs from the memorization phase or not, with corrective feedback (right/wrong). This phase contained the six memorized bugs and six of the other bugs. This part of the recognition phase continued until the participant had reached 100% correct on a block of 6 × 2 bugs. In a second part of the recognition phase, participants were shown each of the six bugs from the memorization phase together with a toxicity value, in random order, and were asked whether it was the correct toxicity value or not (feedback: right/wrong). This phase contained the six memorized bugs with their correct toxicity value and the same bugs but with an incorrect toxicity value. This part of the recognition phase also continued until the participant had reached 100% correct on a block of 6 × 2 bugs. In the *judgment training phase*, participants were taught how to use the Death bugs they had just learnt, in order to judge the toxicity of other bugs. Participants were instructed that upon viewing a bug they were supposed to consider whether they had memorized any bug(s) that were similar to the one they were shown currently and to consider the toxicity value of that/those bugs, in order to come up with a toxicity estimate between 0 and 30. Participants were then given a few practice trials (without feedback) for getting acquainted with making judgments about the toxicity with the newly learnt knowledge. On some practice trials, the shown bug was only similar to one of the memorized bugs (for a separate manipulation, not reported). Participants were also given a few practice trials for the cognitive-perceptual baseline task: to judge whether similarly looking bugs had a gray dot painted on the bottom, top or both bottom and top of the body. Finally, in the *repetition phase*, participants were shown all six memorized bugs again together with their toxicity value, in a random order on the screen, for 8 s each, in order to make sure they would remember them correctly.

Next, participants were invited to the scanner for a test phase (see below under *Imaging procedure*).

### Spontaneous Strategies

In a between-subject design, the task was to judge the toxicity of the Death bugs (Table 2). To induce spontaneous use of rule-based processing or similarity-based processing, respectively, we used a task manipulation previously shown to be effective for this goal (see e.g., Karlsson et al., 2007; Juslin et al., 2008; von Helversen and Rieskamp, 2009; Hoffmann et al., 2013). It has been demonstrated that if the cue-combination rule that combines the cue values into a criterion is additive, participants can abstract the linear relations between each cue and the criterion, and prefer a rule-based strategy. However, if instead the cue-combination rule is multiplicative, participants are unable to abstract the independent contribution of each cue to the criterion. In such a task, participants have instead been shown to prefer a similarity-based strategy (see e.g., Karlsson et al., 2007; Juslin et al., 2008; von Helversen and Rieskamp, 2009; Hoffmann et al., 2013). Half of the participants in the spontaneous strategy condition learnt to make judgments with outcome feedback in an *additive task* and half in a *multiplicative task* (Table 2).

**Table 2:** Items in the spontaneous strategies conditions (experiment 2).

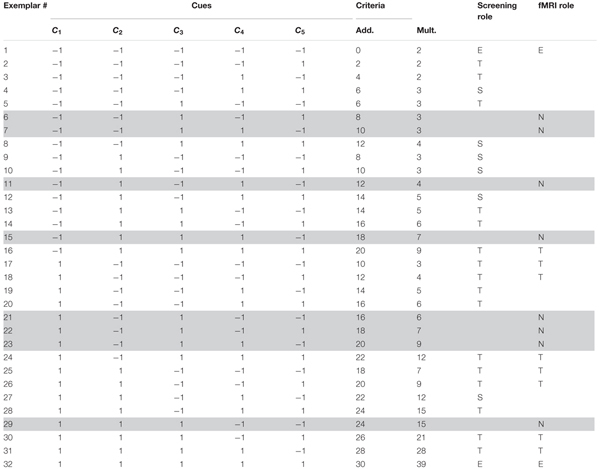

*The table shows the cues and criterion values of all exemplars, and the exemplars that were used during the screening procedure and the fMRI test, respectively. Add., Additive criterion; Mult., Multiplicative criterion; T, exemplars included during the training phase and during the screening test and/or fMRI test, for both conditions; S, new exemplars included only during the screening test for both conditions; N, new exemplars included only during the fMRI test for both conditions; E, extreme exemplars included during the screening test and the fMRI test for both conditions, but used only for behavioral measures. Marked in gray are items critical for the reported fMRI contrast analyses.*

The general procedure for the spontaneous strategy conditions was as follows: in a *screening training phase*, participants judged the toxicity of illustrations of Death bugs, and received outcome feedback after each trial in the form of the correct numerical answer. The training phase continued until a performance criterion was met, or after a maximum of 640 trials. After the training phase followed a *screening test phase*, where judgments were made without outcome feedback. Participants best fit by *CAM* in the additive task and by *EBM* in the multiplicative task, were sent to do a second test phase during fMRI later the same day.

#### Participants

The 137 neurologically healthy participants, with normal or corrected-to-normal vision, were recruited for spontaneous strategy adoption, whereof the 41 that were best fit by *CAM* in the additive task and *EBM* in the multiplicative task were sent to the fMRI session (*CAM* adoption, *n* = 20; *EBM* adoption, *n* = 21; *M*_age_ = 23.3; range = 19 to 37; *SD*_age_ = 3.5; 16 females). See below under *Cognitive modeling* for details regarding the modeling. Participants received 700 SEK for participation. We discarded data from five participants from the analyses: one because of extensive head movements, two because they were ruled out during the screening procedure but mistakenly sent to the scanner, one misinterpreted the instructions during fMRI testing and one because the cognitive modeling of the fMRI test data suggested he or she was not using either *CAM* or *EBM*.

#### Judgment Task

As for the instructed conditions, the Death bugs varied on the same five binary cues (i.e., legs, eyes, back, head and mandibles) with cue values “-1” or “1”. The criterion values (the toxicity, *c*) in the spontaneous strategy conditions were given by either a linear, additive function or a multiplicative function of the five cues (see also Juslin et al., 2008). In the additive condition, the toxicity, *c*, is:

(2) $$c=15+5\times C_{1}+4\times C_{2}+3\times C_{3}+2\times C_{4}+1\times C_{5}.\,$$

In the multiplicative condition, the toxicity, *c*, is:

(3) $$c=2+3\times e^{(5\times C_{1}+4\times C_{2}+3\times C_{3}+2\times C_{4}+1\times C_{5})/6}$$

The training range was held constant between the additive and multiplicative conditions, such that the bug with the lowest and highest criterion values encountered during the training phase had the same value for both conditions (see Table 2).

#### Behavioral Procedure

Participants were randomly assigned to the additive or the multiplicative task condition. First, participants read a cover story about the species of the Death bugs and were informed that Death bugs have varying toxicity. Next, during the *screening training phase*, illustrations of the Death bugs were presented one at a time on a computer screen, and participants made their judgments by typing the toxicity of each Death bug as a numerical estimate between 0 and 100 (%) on the keyboard, immediately followed by the correct numerical feedback. The training phase consisted of 16 different exemplars (exemplars denoted “T” in Table 2) per block. The training phase continued until a learning criterion was met (<2 RMSE between criterion and judgment on one block) or after a maximum of 40 blocks (640 trials). After the training phase followed a *screening test phase*. Participants judged 24 exemplars twice without outcome feedback. The screening test phase consisted of the 16 training exemplars, six new exemplars (exemplars denoted “S” in Table 2) and the two extreme exemplars (denoted “E” in Table 2). The screening test phase was used to analyze which participants were best fit by *CAM* or *EBM*. Participants best fit by *CAM* in the additive condition and by *EBM* in the multiplicative condition were invited to an fMRI test phase later the same day (see below under *Imaging procedure*).

### Imaging Procedure, Image Acquisition, and Data Analyses

#### Imaging Procedure

**FIGURE 2:**
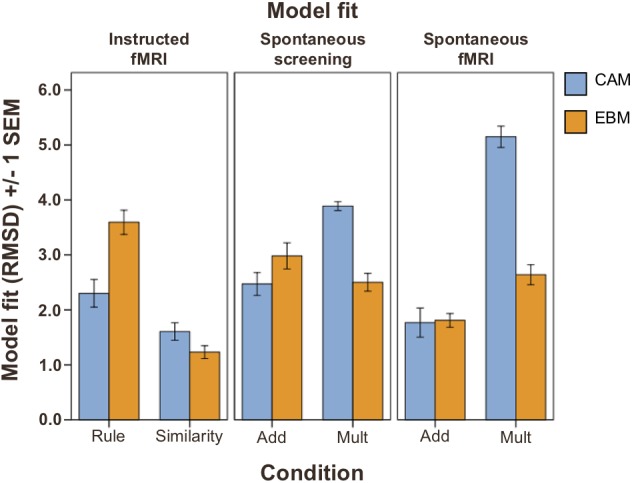
Model fit (RMSD; lower values indicate better model fit) on test phase judgment data (Total *n* = 74). **Left panel**: instructed strategies conditions (*n* = 38); **middle and right panel**: spontaneous strategies conditions (*n* = 36). Blue bars: fit of the *CAM* model. Orange bars: fit of the *EBM* model. Rule, instructed to use rule-based strategy. Similarity, instructed to use similarity-based strategy. ADD, additive learning environment inducing rule-based processing (see section “Materials and Methods”). MULT, multiplicative learning environment inducing similarity-based processing (see section “Materials and Methods”). Error bars denote ±1 SEM.

E-prime 2.0 (Psychology Software Tools, Inc., United States) was used to control presentation and logging of responses, and Lumitouch fMRI optical response keypads (Photon Control Inc., Canada) were used to collect responses. During fMRI, participants in both the instructed and spontaneous strategy conditions judged Death bugs and visual detection baseline bugs without outcome feedback (see Figure 2 and Table 1, 2). Each Death bug was judged three times, in random order, for a total of three functional runs. Each baseline bug was however unique, so the baseline bugs were not repeated across the test. After a task probe (1.5 s.), participants had a maximum of 20 s. (self-paced) to indicate with their right ring finger on a four-button keypad when they had formed a judgment. This was followed by a jittered crosshair (2–10 s.) and a numerical response scale (self-paced, max. 10 s.), which participants could step through to give their response (step to the left on the scale: right index finger, step to the right: the right middle finger, confirm: right ring finger). After a last jittered cross-hair (2–6 s.) the next trial began.

#### Image Acquisition and Preprocessing

All images were acquired using a 3.0 T whole-body MRI system (MR 750, GE, Medical Systems) equipped with a 32 channels head coil. T2^∗^-weighted images were obtained with a single-shot GE-EPI sequence used for BOLD imaging. The sequence had the following parameters: echo time (TE) 30 ms; repetition time (TR) 2000 ms; flip angle 80°; field of view (FOV) 25 cm; matrix = 96 × 96; 3.4 mm slice thickness (37 slices acquired). The 10 dummy scans were collected prior to data collection to allow for equilibration of the fMRI signal. High-resolution T1-weighted structural images were also obtained for each participant. Stimuli were presented on a computer screen seen by the participant through a mirror attached to the head coil. To reduce perception of the scanner noise, headphones and earplugs were used, and cushions in the coil reduced head movement.

Functional data were preprocessed and analyzed in SPM8 (The Wellcome Department of Cognitive Neurology, London, United Kingdom), with a batch function in an in-house program (*DataZ*). All images were corrected for slice timing, realigned with unwarp to correct for head movements, spatially normalized to MNI-space and smoothed (8 mm FWHM Gaussian filter kernel). Statistical analyses were calculated on the smoothed data with a high-pass filter (128 s cutoff period) in order to remove low-frequency noise.

#### fMRI Data Analyses

As judgments on new test items are most diagnostic concerning the cognitive processes in question, and, importantly, as the new test items shared the same history for all conditions (i.e., they had never been encountered before during instructions or training) the analyses of fMRI data concentrated on the contrasts between judgments on new test items (denoted with “N” in Tables 1, 2) and judgments of baseline bugs. Thus, as effects of interest were the task events of new death bugs and baseline bugs, respectively. For the instructed strategy conditions, three effects of no interest were modeled: task events with bugs with only two cues visible/bugs with only one similar exemplar (for a separate manipulation not reported here), task events with memorized exemplars (denoted with “M” in Table 1) and the rating scale events. For the spontaneous strategy conditions, two effects of no interest were modeled: task events with training exemplars (denoted with “T” in Table 2) and the rating scale events. The six movement parameters were included as covariates of no interest.

All regressors except the head movement parameters were convolved with a hemodynamic response function. In the first level analysis, model estimations were made for each participant and contrast images (“N” items vs. baseline items) were generated for input to the second-level analyses. Second-level analyses consisted of three main types: (*i*) two-sample *t-*tests to investigate activation differences between the conditions, (*ii*) conjunction analyses to investigate whether the conditions shared brain activity (testing the “conjunction null” hypothesis; Nichols et al., 2005), and (*iii*) correlational analyses (Pearson’s *r*) with model fit (RMSD for *CAM* and *EBM*) to investigate the potential functional roles played by such shared brain activity.

Three conjunction analyses were made: one across all four rule-based and similarity-based conditions, one across the two rule-based conditions and one across the two similarity-based conditions.

A set of correlational analyses were carried out between model fit and brain activity across the whole sample (*n* = 74). For these analyses, brain activity was averaged across voxels within a sphere with a radius of 5 mm around peak voxels from the conjunction analyses. First, correlation analyses were made between model fit (RMSD for the *CAM* and *EBM* model, respectively) and activity in brain regions identified with the conjunction analysis with all four rule-based and similarity-based conditions. Second, correlation analyses were made between model fit and activity in brain regions identified with the conjunction analysis with the two rule-based conditions. Third, correlation analyses were made between model fit and activity in brain regions identified with the conjunction analysis with the two similarity-based conditions. We test whether the difference between correlations is significant by an asymptotic *z*-test (Lee and Preacher, 2013).

The statistical threshold for contrasts and conjunction analyses was set to *p* < 0.05 (FDR-corrected at the voxel level), for the correlation analyses it was set to *p* ≤ 0.05 (Bonferroni correction will be used) and for the asymptotic *z*-test it was set to *p* ≤ 0.05.

Technically, the conjunction analyses were conducted as follows: for each of the individual group tests considered in the conjunction analyses, an uncorrected *p*-value was calculated for each voxel. A conjunction was made by taking the maximum of these *p*-values for each voxel, using a function in the in-house program (*DataZ*). The same method as implemented in SPM was then used to calculate FDR-corrected *p*-values.

One set of control analyses was carried out. Following the correlation analyses, two whole-brain correlation analyses were made, in order to investigate the specificity of any of the functional relations identified in the first correlation analyses. To accommodate this, we set up two general linear models using task vs. baseline with model fit of *EBM* and model fit of *CAM*, respectively, as covariates of interest (*p* < 0.05 FDR-corrected).

#### Cognitive Modeling

We used mathematical models to infer which strategy a participant was relying on.

##### Cue abstraction model

The cue abstraction model (*CAM*) assumes an abstracted knowledge of the cue-criterion relations which connect a specific cue to a criterion (Juslin et al., 2003). The final estimate 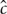
_CAM_ is a linear additive function of the cues C_i_,

(4) c^CAM=k+Σi=15ωi⋅Ci

where the intercept (*k*) and the cue-weights (*w_i_*) are free parameters (see also Juslin et al., 2008).

##### Exemplar-based model

The *EBM* assumes judgments are based on the memory of previously encountered exemplars (Juslin et al., 2003). The final estimate 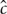
_EBM_ is continuous and is given by

(5) c^EBM=ΣNSn⋅cnΣNSn,

where *N* refers to the number of exemplars, *S*_n_ refers to the probe-exemplar similarity and *c*_n_ to the criterion of exemplar *n*. The similarity rule of the original context model (Medin and Schaffer, 1978) was applied to compute the similarity between the probe and exemplar*x*_n_,

(6) $$S(n)=\Pi_{i}^{I}d_{i}$$

where *d_i_* is an index that takes value 1 if the cue values on cue dimension *i* (i = 1,…, I) coincide and *s_i_* if they deviate. *s_i_* are five free parameters in the interval [0, 1] (see also Juslin et al., 2008). When using *EBM* to predict judgments in the instructed rule-based condition, for simplicity, an identical pool of six previously encountered exemplars was assumed as for the instructed similarity-based condition.

The models were fitted to each participant’s individual judgment data from the fMRI judgment test phases with a leave-one-out cross validation procedure (Stone, 1974). The free parameters of the models were estimated by fitting the models to all but one of the test items to predict the response of the remaining test item with the estimated parameters. This procedure was repeated for all test items. Root mean squared deviation (RMSD) between the models predictions and the participants judgments were used as a goodness-of-fit criterion. The parameters were estimated using unconstrained non-linear optimization with a *simplex* algorithm as implemented in MATLAB (MathWorks Inc., Natick, MA, United States).

For cognitive modeling of the screening test-phase judgments in the spontaneous conditions, the models were fitted to each participant’s individual data with a procedure of *projective fit* (Juslin et al., 2003). Parameters for each of the models were estimated from the last part of the training phase (i.e., the last three blocks) and the best fitting parameters were used to predict the screening test phase data. RMSD between the models predictions, and participants judgments were used as a goodness-of-fit criterion. The parameter values for *EBM* were estimated with a non-linear least squares fit as implemented in MATLAB (MathWorks Inc., Natick, MA, United States). The parameter values for *CAM* were estimated analytically with multiple linear regression (as implemented in MATLAB).

## Results

### Performance

*Performance* was measured as the root mean squared error (RMSE) between participants’ judgment data and criteria. All participants included in the analyses (*n* = 74) reached the learning criteria by passing the instruction procedure in the instructed strategy conditions or, in the spontaneous strategy conditions, by having a RMSE < 2 on one block of training items before reaching 40 blocks.

Performance (RMSE) on the test phase items critical for the fMRI contrast analyses (i.e., new items introduced at test, denoted “N” in Tables 1, 2) is presented in Table 3. Two one-way ANOVAs with strategy condition (rule-based vs. similarity-based) as between-subjects factor and performance (RMSE) as dependent variable revealed an effect of strategy condition for both learning modes: instructed strategies (*F*_(1,36)_ = 4.87; *MSE* = 12.2; *p* = 0.034) and spontaneous strategies (*F*_(1,34)_ = 11.3; *MSE* = 29.5; *p* = 0.002). Participants on average performed better on these test phase items in the rule-based conditions compared to the similarity-based conditions.

**Table 3:** Mean performance (RMSE) on test items critical for the fMRI contrast analyses (i.e., new items introduced at test, denoted “N” in Tables 1, 2) separately for the four conditions.

	Strategy condition	
	
Learning mode	Rule	Similarity
Instructed	4.26 (1.89)	5.39 (1.20)
Spontaneous	2.62 (0.96)	4.45 (1.99)


*Numbers within parentheses are standard deviations.*

Importantly, the test phase was designed to produce qualitatively distinct response patterns with rule-based and similarity-based processes, predicted by the *CAM* model and the *EBM* model, respectively. Therefore, the key analysis of our behavioral data is to use cognitive modeling to infer which model fit participants’ data best.

### Cognitive Modeling Evidence for Similarity-Based and Rule-Based Judgment Processes

The *model fits* (as estimated with cross-validation and measured with RMSD, between predictions and test phase judgment data) demonstrated that participants in the similarity-based conditions were better fit by the *EBM* model than by the *CAM* model, while in the rule-based conditions, participants were better fit by the *CAM* model, or equally well fit by both models (Figure 2, see Supplementary Table S1 for mean best fitting parameter values). Repeated measurements ANOVAs with model fit (*CAM* vs. *EBM*) as within-subjects factor and condition (inducing rule-based or similarity-based processing) as between-subjects factor revealed a significant interaction between model and condition for both learning modes: instructed strategies (*F*_(1,36)_ = 48.8; *MSE* = 13.2; *p* < 0.001) and spontaneous strategies (in the screening test phase before the fMRI session: *F*_(1,34)_ = 84.6; *MSE* = 15.9; *p* < 0.001; in the fMRI judgment test phase: *F*_(1,34)_ = 53.3; *MSE* = 30.7; *p* < 0.001).

### Direct Contrasts Between Rule-Based and Similarity-Based Judgments

For the fMRI data, for being able to explicitly relate the brain imaging results to the related literature on the topic focusing on activation *differences* between the two kinds of processes, we first report activation differences (see Table 4 for a list of significant clusters).

**Table 4:** Two-sample *t*-tests on fMRI-data.

*t*-tests	Cluster #	Region	BA	*x*	*y*	*z*	*t*-value	Voxels (*k*)
Instructed *EBM* vs.								
Instructed *CAM*								
	1	Precuneus	23/31/18	-6	-60	26	5.31	304
	2	Superior frontal gyrus/sulcus	10/46	-22	56	20	5.26	271
	3	Medial superior frontal gyrus	8	-2	32	56	4.82	228
	4	Angular gyrus	19/39	-42	-78	40	4.22	27
	5	White matter		-12	58	0	4.10	17
	6	Cuneus	19	2	-84	32	4.06	8
	7	Anterior cingulate cortex	32	-8	38	26	3.85	2
Spontaneous *CAM* vs.								
Spontaneous *EBM*								
	1	White matter		28	-58	24	5.06	5799
		Central sulcus	4/1	62	-4	22	3.95	
		Middle frontal gyrus	46	42	46	14	3.40	
	2	White matter		-32	-18	30	5.02	14481
		Superior parietal	7	-26	-52	58	4.88	
		Precentral sulcus	6	-36	-6	64	4.61	
		Superior parietal	7	24	-60	58	4.33	
		Middle frontal gyrus	8	-32	36	40	4.14	
	3	Superior temporal sulcus	21	-56	-28	-4	4.19	224
	4	Putamen		20	0	-12	4.02	348
	5	Hippocampus		-32	-26	-10	3.96	244
	6	Cerebellum		-12	-46	-16	3.89	1241
	7	White matter		-4	2	8	3.66	164
	8	Cerebellum		38	-52	-40	3.62	135
	9	Medial superior frontal gyrus	6	-2	-6	72	3.61	164
	10	Hippocampus		36	-30	-6	3.43	65
	11	Precentral sulcus	6	26	-8	74	3.39	45
	12	White matter		-50	2	-14	3.25	58
	13	Cerebellum		-42	-66	-34	3.21	99
	14	Superior temporal sulcus	21	52	-4	-10	3.21	30
	15	White matter		-28	-58	-42	3.05	56
	16	White matter		-26	-44	-38	2.93	15
	17	Cerebellum		-18	-88	-26	2.91	7
	18	White matter		-16	54	18	2.89	43
	19	Insula		-34	4	-4	2.87	29
	20	Brain stem		-8	-32	-4	2.84	14
	21	Cerebellum		-2	-78	-38	2.84	27
	22	Insula		40	0	-8	2.79	47
	23	Parahippocampal gyrus	35	14	-38	-8	2.77	5
	24	Cingulate sulcus	32	0	6	44	2.74	21
	25	Brain stem		-4	-22	-2	2.71	2
	26	Middle temporal gyrus	22	66	-38	10	2.70	4
	27	Supramarginal gyrus	40	64	-30	32	2.70	10
	28	Superior temporal sulcus	21	54	6	-14	2.65	4
	29	White matter		44	-34	40	2.64	13
	30	Inferior temporal gyrus	37	52	-58	-20	2.58	1
	31	White matter		32	-46	-12	2.58	1
	32	Brain stem		6	-22	-4	2.58	1


*Up to 5 local maxima are reported for each cluster. BA, Brodmann area. Coordinates (*x*, *y*, *z*) in MNI space (SPM8). *t*-values at the peak voxel. Voxel: *p <* 0.05 (FDR-corrected). Cluster: *k* > 0.*

One previous study has investigated the neural correlates of rule-based and similarity-based multiple-cue judgments by contrasting brain activity during an instructed similarity-based process with an instructed simple heuristic rule-based process (von Helversen et al., 2014b). Even though the heuristic process instructed in that study can be assumed to be less cognitively demanding than the rule-based processes under study here, it might still be worth-while to relate the two studies. In line with von Helversen et al. (2014b), for instructed similarity-based processing compared to instructed rule-based processing we observed higher brain activity in left anterior prefrontal cortex (BA 10/46) and left inferior parietal cortex (angular gyrus). In addition, we observed higher brain activity in precuneus, cuneus and anterior cingulate cortex for instructed similarity-based processing compared to instructed rule-based processing. For instructed rule-based processing compared to instructed similarity-based processing von Helversen et al. (2014b) identified a set of clusters, including regions in bilateral superior frontal cortex (BA6/4) and left supramarginal gyrus. We observed no significant differences comparing instructed rule-based processing with instructed similarity-based processing under the chosen statistical threshold. It should be noted though, that under a less conservative threshold (*p* < 0.001, uncorrected) we were also able to identify superior frontal cortex (BA6) and left supramarginal gyrus in very close proximity to the peaks reported by von Helversen et al. (2014b): bilateral precentral sulcus (MNI *x, y, z* coordinates: 28, -14, 64; *t* = 4.42; *p* < 0.001; -28, -16, 56; *t* = 4.19, *p* < 0.001), superior frontal gyrus (BA6: MNI *x, y, z* coordinates: 12, -10, 64; *t* = 4.16; *p* < 0.001) and left supramarginal gyrus (BA40: MNI *x, y, z* coordinates: -60, -46, 34; *t* = 3.90; *p* < 0.001). Taken together, the results comparing instructed versions of the strategies also bear many similarities with corresponding studies from the categorization literature (e.g., Patalano et al., 2001; Koenig et al., 2005).

Next, we compared brain activity for the spontaneous versions of the strategies. For spontaneous rule-based processing compared to spontaneous similarity-based processing, brain activity was higher in a large number of clusters including precentral sulcus and superior frontal gyrus (BA6), supramarginal gyrus, bilateral superior parietal cortex, as well as left and right middle frontal gyrus, superior temporal sulcus, putamen, hippocampus and cerebellum (Table 4). However, spontaneous similarity-based processing compared to spontaneous rule-based processing revealed no clusters active above the statistical threshold. This was the case even under a less conservative threshold (*p* < 0.001, uncorrected), implying that the two processes might share important components.

### Analyses of Overlap in Brain Activity Implicate the Precuneus in All Judgment Conditions

**FIGURE 3:**
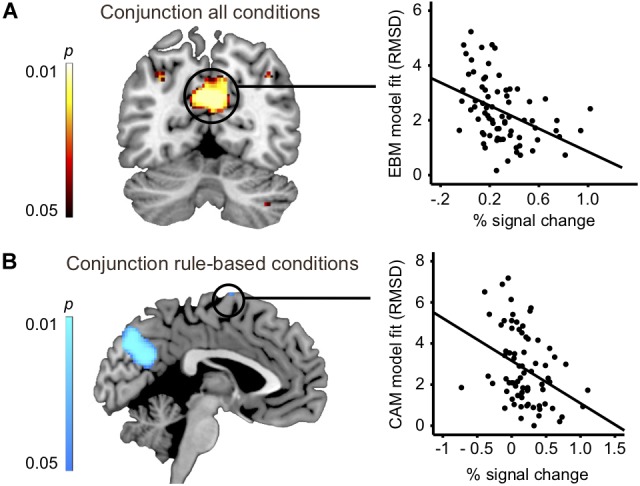
**(A)** Left panel: conjunction analysis: all four conditions vs. baseline (slice at MNI coordinate *y* = –60). Right panel: Correlation analysis: Brain activity difference between task and baseline in precuneus (Table 5) significantly correlated with model fit of the exemplar-based model (RMSD: *r* = –0.42; *p* < 0.001; *n* = 74). **(B)** Left panel: Conjunction analysis: instructed and spontaneous rule-based conditions vs. baseline (slice at MNI coordinate *x* = –2). Correlation analysis: Brain activity difference between task and baseline in superior frontal gyrus (BA6, Table 5) significantly correlated with model fit of the cue-abstraction model (RMSD: *r* = –0.36; *p* = 0.002; *n* = 74).

To address the issue of whether the relationship between rule-based and similarity-based processes should be construed according to an *either-or* view or an *interplay* view, we analyzed the overlap in task-related brain activity. A conjunction analysis between all four rule-based and similarity-based conditions revealed that the conditions shared considerable regional activity in a set of areas, namely: precuneus, bilateral inferior parietal cortex (angular gyrus) and cerebellum, suggesting that rule-based and similarity-based processes share important processing components (Figure 3A and Table 5).

**Table 5:** Conjunction analyses on fMRI data.

Conjunction	Cluster #	Region	BA	*x*	*y*	*z*	*p*-value	Voxels (*k*)
All four conditions								
	1	Precuneus	23/31/18	-10	-60	16	0.013	1182
	2	Angular gyrus	39	-32	-72	42	0.013	136
	3	Angular gyrus	39	38	-64	40	0.024	15
	4	Cerebellum		36	-66	-42	0.038	4
Instructed and								
spontaneous *EBM*								
	1	Precuneus	23	-6	-66	24	0.00016	6147
		Retrosplenium	26	6	-40	24	0.00049	
		Calcarine	31	2	-56	16	0.0005	
	2	Angular gyrus	39	-34	-74	44	0.00016	706
	3	Angular gyrus	7	38	-68	44	0.0027	191
	4	Inferior frontal sulcus	46	-42	44	6	0.0031	205
	5	Cerebellum		40	-64	-42	0.0056	70
	6	Middle frontal gyrus	9	48	32	24	0.008	42
	7	White matter		-56	-50	-16	0.013	16
	8	Cerebellum		12	-50	-20	0.016	49
	9	Medial superior frontal gyrus	6	-8	10	52	0.022	21
	10	Superior frontal gyrus	10	-22	62	8	0.025	15
	11	Inferior frontal sulcus	46	-42	24	26	0.031	55
	12	middle frontal gyrus	6	-30	4	62	0.041	4
	13	Lingual gyrus	18	-8	-80	0	0.042	11
	14	Medial superior frontal gyrus	6	-4	26	40	0.043	2
	15	Central sulcus	3	-32	-28	62	0.046	1
Instructed and								
spontaneous *CAM*								
	1	Precuneus	23/31/18	-12	-60	18	0.01	1363
	2	Angular gyrus	39	-32	-76	46	0.01	164
	3	Precentral sulcus	44	-48	12	36	0.015	68
	4	Cerebellum		32	-62	-44	0.015	70
	5	Angular gyrus	39	38	-64	38	0.019	39
	6	Inferior occipital gyrus	17	-6	-102	-2	0.024	17
	7	Middle frontal gyrus	9	48	30	32	0.039	6
	8	Medial superior frontal gyrus	6	-2	-2	74	0.042	2
	9	Intraparietal sulcus	7	-34	-54	36	0.05	1


*Up to 5 local maxima are reported for each cluster. BA, Brodmann area. Coordinates (*x*, *y*, *z*) in MNI space (SPM8). *p*-values at the peak voxel. Voxel: *p <* 0.05 (FDR-corrected). Cluster: *k* > 0.*

Two additional conjunction analyses were made. First, a conjunction analysis with the two rule-based conditions (instructed and spontaneous) revealed an almost identical pattern as when all four conditions were included, and in addition revealed shared brain activity in dorsolateral prefrontal cortex (precentral sulcus, BA44), inferior occipital gyrus, middle frontal gyrus, superior frontal gyrus (BA6) and intraparietal sulcus (Figure 3B and Table 5). Second, a conjunction analysis with the two similarity-based conditions (instructed and spontaneous) also revealed an almost identical pattern as when all four conditions were included (Table 5), along with shared brain activity in several frontal clusters, such as inferior frontal sulcus, middle frontal gyrus, and superior frontal gyrus, as well as in lingual gyrus.

### Precuneus Activity Was Functionally Related to Similarity-Based Judgments

Does any of the brain regions identified in the conjunction analysis between all four conditions play a functional role for judgment, in the sense of being related to model fit? We related brain activity in the identified regions in the conjunction analysis between all four conditions (four significant clusters: Figure 3A and Table 5) with model fit (RMSD for *CAM* and *EBM*, respectively). Because two hypotheses were tested per cluster (i.e., the relation to *CAM* and *EBM*) the Bonferroni-corrected significance threshold for each correlation analysis was *p* < 0.006. We observed a significant correlation between model fit of *EBM* and brain activity differences in the strongest cluster in the conjunction – the precuneus – such that the better *EBM* predicted participants data (i.e., the smaller the RMSD), the higher was the activity difference between the judgment task and the baseline task in the precuneus (*r* = -0.42; *p* < 0.001; *n* = 74: Figure 3A). The correlation with the fit of *CAM* in this region was low and non-significant (*r* = -0.12; *p* = 0.31; *n* = 74). The difference between these two correlations was significant (*r* = -0.42 vs. *r* = -0.12; *z* = -2.4; *p* = 0.008). We observed no correlations above statistical threshold between model fits of *CAM* or *EBM* and activity differences in any of the other three clusters that were observed in the conjunction analysis between all four conditions (i.e., bilateral inferior parietal cortices or cerebellum; Table 5).

To assess the robustness of the observed correlation between shared activity in precuneus and model fit of *EBM* we conducted two additional sets of analyses. First, we correlated model fit of *EBM* and *CAM*, respectively, with activity difference in each of the remaining six local maxima in the precuneus cluster (yielding a Bonferroni-corrected threshold of *p* < 0.004). Four of these six correlations were significant (*r* = -0.38, *p* = 0.001; *r* = -0.43, *p* < 0.001; *r* = -0.40, *p* < 0.001; *r* = -0.38, *p* < 0.001; *n* = 74). The correlations with the fit of *CAM* in these four local maxima were low and non-significant (*r* = -0.07; *p* = 0.57; *r* = -0.13; *p* = 0.27; *r* = -0.01; *p* = 0.41; *r* = -0.28; *p* = 0.02; *n* = 74).

Second, we examined to what extent the observed correlation between shared activity in precuneus and model fit of *EBM* holds for each of the four separate conditions, in order to control that the overall correlation was not driven exclusively by the conditions using a similarity-based process. Due to the small sample size in each condition (16 ≤*n* ≤ 19), the correlations between activity difference in precuneus and model fit of *EBM* were small and non-significant. Importantly, there was a similar trend in all four conditions, with a negative correlation between model fit of *EBM* and activation difference in precuneus (Instructed rule-based: *r* = -0.21, *p* = 0.38, *n* = 19; Instructed similarity-based: *r* = -0.23, *p* = 0.36, *n* = 19; Spontaneous rule-based: *r* = -0.15, *p* = 0.58, *n* = 16; Spontaneous similarity-based: *r* = -0.21, *p* = 0.39, *n* = 20). There were no significant differences between these four correlations: all *p*’s > 0.82 (Preacher, 2002). We interpret these results as that the overall correlation is not driven exclusively by the two groups using a similarity-based process (or the two groups using a rule-based process).

### Superior Frontal Gyrus (BA6) Activity Was Functionally Related to Rule-Based Judgments

The conjunction analysis between the two rule-based conditions identified an almost identical pattern as when all four conditions were included and, in addition, shared brain activity in dorsolateral prefrontal cortex (precentral sulcus, BA44), inferior occipital gyrus, middle frontal gyrus, superior frontal gyrus (BA6) and intraparietal sulcus (see Table 5). Because two hypotheses were tested per each of these additional five clusters the Bonferroni-corrected significance threshold for each correlation analysis was *p* < 0.005. We observed a significant correlation between brain activation difference in superior frontal gyrus (BA6) and model fit of *CAM* (*r* = -0.36; *p* = 0.002; *n* = 74: Figure 3B). Also, we observed a significant correlation between brain activation difference in precentral sulcus (BA44) and model fit of *CAM* (*r* = -0.33; *p* = 0.004; *n* = 74). The correlations between the model fit of *EBM* and activity in these regions were low and non-significant (superior frontal gyrus (BA6): *r* = 0.09; *p* = 0.43; *n* = 74, precentral sulcus (BA44): *r* = -0.19; *p* = 0.10; *n* = 74). However, while the difference between the two correlations was significant for superior frontal gyrus (BA6: *r* = -0.36 vs. *r* = 0.09; *z* = -3.5; *p* < 0.001) it was not significant for precentral sulcus (BA44: *r* = -0.33 vs. *r* = -0.19; *z* = -1.11; *p* = 0.13), questioning the specificity of the precentral sulcus activation difference in relation to *CAM*.

We observed no correlations above statistical threshold between model fits of *CAM* or *EBM* and activity differences in any of the other identified additional clusters.

The conjunction analysis between the two similarity-based conditions identified an almost identical pattern as when all four conditions were included and, in addition, shared brain activity in several frontal clusters, such as inferior frontal sulcus, middle frontal gyrus, and superior frontal gyrus, as well as in lingual gyrus (Table 5). Because two hypotheses were tested per each of these additional nine clusters (the white matter cluster not included) the Bonferroni-corrected significance threshold for each correlation analysis was *p* < 0.003. We observed no correlations above statistical threshold between model fits of *EBM* or *CAM* and activity differences in any of these additional nine clusters.

### Whole-Brain Correlation Analyses Confirmed Specificity of Precuneus and Superior Frontal Gyrus

For completeness, whole-brain correlation analyses were performed in order to investigate to what extent the correlations between activity differences in precuneus and model fit of *EBM*, and activity differences in superior frontal gyrus (BA6) and precentral sulcus (BA44) and model fit of *CAM*, respectively, could be replicated when applying an exploratory perspective (see Supplementary Tables S2, S3).

The results revealed that precuneus was included in the strongest cluster correlating with model fit of *EBM*, thus replicating the negative correlations between activity differences in precuneus and model fit of *EBM.* At the same time, superior frontal gyrus in the vicinity of the cluster identified above as correlating with model fit of *CAM* was not identified in this analysis (under the chosen statistical threshold, Supplementary Table S2). However, precentral sulcus (BA44) overlapped with clusters identified in this analysis (Supplementary Table S2), again questioning its specificity for *CAM*. The correlation with the model fit of *CAM* in the precuneus local maximum was low and non-significant (*r* = -0.06; *p* = 0.62; *n* = 74). Interestingly, among the strongest local maxima correlating with model fit of *EBM* were also peaks in anterior medial prefrontal cortex and bilateral hippocampus (Supplementary Table S2).

In terms of whole-brain correlation analysis with model fit of *CAM*, the negative correlations between activity differences in superior frontal gyrus (BA6) and precentral sulcus (BA44) and model fit of *CAM* was replicated, whereas precuneus in the vicinity of the cluster identified above as correlating with model fit of *EBM* was not identified in this analysis (under the chosen statistical threshold, Supplementary Table S3). The correlation with the model fit of *EBM* in the superior frontal gyrus local maxima of interest (*x, y, z* = 2, -2, 72) was low and non-significant (*r* = 0.09; *p* = 0.47; *n* = 74). Among the strongest local maxima correlating with model fit of *CAM* were also peaks in parieto-occipital cortex, temporo-parietal cortex, precentral sulcus and middle frontal cortex (Supplementary Table S3).

## Discussion

The current study contributes with novel neuroimaging findings related to the distinction between inferences based on analytical processes and inferences based on associative memory (e.g., Patalano et al., 2001; Grossman et al., 2002; Koenig et al., 2005; von Helversen et al., 2014b). We demonstrate that (***i***) when contrasting brain activity during rule-based and similarity-based judgment processes there are observable activation differences in brain regions corroborating previous related literature (Patalano et al., 2001; Koenig et al., 2005; von Helversen et al., 2014b), (***ii)*** both kinds of processes share extensive task-related brain activity, most notably in parietal cortex: specifically in the precuneus, and in bilateral inferior parietal cortices (angular gyrus), (***iii***) task-related brain activity in the precuneus correlates with how well a similarity-based model fit judgment data, while (***iv***) task-related brain activity in superior frontal gyrus (BA6) correlates with how well a rule-based model fit judgment data. These results imply key roles for superior frontal gyrus (BA6) and the precuneus in human judgment, and have implications for how a relationship between the two kinds of processes can be understood. The findings are based on a large sample across four conditions. In particular, the evidence for a role of precuneus in human judgment is considerable, as this region was associated with the strongest effect (in terms of peak activity as well as extent) in all four conditions.

Parietal cortices has been found to be pivotal for judgment, decision making, reasoning and categorization (see e.g., Patalano et al., 2001; Greene et al., 2004; Koenig et al., 2005; Gold and Shadlen, 2007; Heekeren et al., 2008; Freedman and Assad, 2011; Prado et al., 2011; Mack et al., 2013; von Helversen et al., 2014b; Seger et al., 2015). To highlight a few examples, Mack et al. (2013) were able to demonstrate the importance of posterior parietal cortices for exemplar-based processes in categorization, when finding support for an *EBM* in the patterns of brain activation in this region. Seger et al. (2015) established that parietal cortices are important when generalizing category membership for untrained visual stimuli, based on trained stimuli. Brain activation differences in the more medial part of parietal cortex known as precuneus have also been observed in relation to several different aspects of higher cognition including memory-related processing such as retrieval from episodic memory (for an overview, see e.g., Cavanna and Trimble, 2006) including recognition memory (Reber et al., 2002; Dorfel et al., 2009). Precuneus activity is also observed when judgments are based on a recognition heuristic (Volz et al., 2006) as well as when stimuli are freely categorized in accordance with a similarity-based rule (Milton et al., 2009). To our knowledge, this study is the first to explicitly stress such direct links between similarity-based processing captured by an *EBM* and precuneus activity. One reason this has not been stressed to a similar extent in previous studies might be the tendency to focus mainly on the brain activity that *differ* between the two types of processes, where precuneus activity is likely to have been canceled out (see e.g., Koenig et al., 2005; von Helversen et al., 2014b).

Tentatively, what could be the role for precuneus in exemplar-based processing? It has been demonstrated that ventral precuneus, together with regions such as medial prefrontal cortex and medial temporal lobes, constitutes a network of regions enabling retrieval from episodic memory (see e.g., Wagner et al., 2005; Vincent et al., 2006). Recently, medial parietal cortex extending down to retrosplenial cortex, has been suggested to support episodic memory by acting as a gateway between medial temporal lobes and other cortical regions (Kaboodvand et al., 2018). Several memory tasks evoking precuneus activation seem to have in common that memory representations need not only be retrieved but also inspected in some way, e.g., with regard to the source, context or spatial details of the representation (e.g., Lundstrom et al., 2005; see also the review by Cavanna and Trimble, 2006). Wagner et al. (2005) explicitly suggested that medial and lateral posterior parietal cortices role in episodic memory might be related to directing attention to internal representations, and/or related to the storage of retrieved memories dynamically in order to make them accessible to decision making. Notably, activity differences in medial prefrontal cortex and hippocampus both fell out as negatively correlated with model fit of *EBM* in our whole-brain correlation analysis (in addition to ventral precuneus, see Supplementary Table S2), indicating that the higher the activity difference also in these regions, the better model fit with *EBM*. One possibility is thus that the key role of precuneus in the task presented here is to aid retrieval and inspection of exemplars from memory, thanks to the link of this part of the brain to the medial temporal lobes and prefrontal cortices. Importantly, this tentative explanation would naturally connect the precuneus findings in our study to several attempts in the categorization literature highlighting an important role for the medial temporal lobes (see e.g., Love and Gureckis, 2007; Seger and Miller, 2010; Davis et al., 2012a,b; Davis and Poldrack, 2014).

Superior frontal gyrus (BA6) has previously been associated with spatial working memory (Tanaka et al., 2005) and mental arithmetic (for an overview, see Zamarian et al., 2009), but also with judgment, decision making, rule-based categorization and rule use (e.g., Patalano et al., 2001; Wallis and Miller, 2003; Koenig et al., 2005; Cisek, 2006; Helie et al., 2010; Klaes et al., 2011; von Helversen et al., 2014b). For example, this region was observed when contrasting brain activation during an instructed rule-based heuristic judgment strategy with activation during an instructed exemplar-based strategy (von Helversen et al., 2014b) as well as when contrasting instructed rule-based and similarity-based strategies in categorization tasks (Patalano et al., 2001; Koenig et al., 2005). Here, this region was associated with rule-based processing both when directly contrasting rule-based to similarity-based judgments, in the conjunction analysis between the two rule-based conditions (instructed and spontaneous), and when investigating its functional role in correlation analyses with model fit of *CAM*.

Taken together, our results lend support to a distinction between rule-based and similarity-based processes at a neural level not only in categorization (Patalano et al., 2001; Grossman et al., 2002; Koenig et al., 2005) but also in human judgment (see also von Helversen et al., 2014b). Critically, our results raise the possibility that the relationship between associative and analytical judgments should not be conceived of as a strict dichotomy where the processes are recruited in an *either-or* fashion (Juslin et al., 2003, 2008; Karlsson et al., 2007, 2008; von Helversen and Rieskamp, 2009; von Helversen et al., 2010, 2013; Mata et al., 2012; Pachur and Olsson, 2012; Hoffmann et al., 2013, 2014).

Instead, our results appear to favor an *interplay* view on how a relationship between the two kinds of processes could be understood. We observed shared brain activity between all four conditions in precuneus, a region extensively linked to memory-based processes, and this activity was predicted by a similarity-based model. This suggest that similarity-based processes are routinely engaged for judgments, *both* when overt responses appear to be guided by similarity-based processes and rule-based processes. Moreover, the observation that only the two rule-based conditions shared activity in superior frontal cortex can be taken to suggest that rule-based processes add on to similarity-based processes under certain circumstances instead of being routinely engaged in parallel to similarity-based processes (see Palmeri, 1997; Ashby et al., 1998; Erickson and Kruschke, 1998; Rieskamp and Otto, 2006). In that latter case, we should have observed that all four conditions shared brain activity in *both* precuneus and superior frontal gyrus (BA6), and that this activity was related to model fit.

Recently, a couple of brain imaging studies corroborate our findings in calling into question the *either-or* view, by likewise considering brain activation overlap with conjunction analyses between conditions (see e.g., Milton and Pothos, 2011; Mega et al., 2015; Carpenter et al., 2016; Milton et al., 2017). Milton et al. (2017) used a category learning design that instead of cognitive modeling enabled analysis of how critical trials were categorized in order to infer whether a participant had learnt the task via similarity- or rule-based processes. The authors were not able to identify any brain activation differences between the strategies in whole-brain comparisons, but rather observed extensive overlap in several brain regions. Milton and Pothos (2011) and Carpenter et al. (2016) were able to demonstrate that two categorization tasks (rule-based vs. information integration tasks) previously believed to recruit two distinct cognitive “systems” (a verbal system vs. a procedural system, cf. Ashby et al., 1998) in fact shared considerable brain activity in brain areas previously assumed to be exclusive to the verbal system. Mega et al. (2015) demonstrated that intuitive and deliberate strategies with which to evaluate emotional facial expressions also shared considerable brain activity to a large extent. As a side note, several of these studies have actually reported precuneus activation in tables or in text, without giving that observation much attention in the paper, such as for example the observation that precuneus activation was significantly shared between the two types of processes (Milton et al., 2017), that precuneus was more engaged with the information integration task than the rule-based task (Carpenter et al., 2016), and that precuneus activation was associated with an intuitive judgment strategy (Mega et al., 2015). The same goes for two very recent and intriguing studies on categorization (e.g., Davis et al., 2017; Bowman and Zeithamova, 2018) both reporting relations between quantitative measures from an *EBM* and activation in precuneus.

Do our results have implications for the more general “dual system” theories, where the distinction between two different processing modes is intensely debated (Sloman, 1996; Stanovich, 1999; Kahneman and Frederick, 2002; Evans and Stanovich, 2013; De Neys, 2018)? Our results suggest how brain imaging methods can be used to test neurocognitive assertions that are difficult to test with behavioral data alone. For example, within some dual systems accounts, the relationship between two different processing modes is described as *default interventionism* where one process acts as default and the other process intervenes (Evans and Stanovich, 2013). In other accounts, the relationship is described as *parallel competitive*, in the sense that both processes are always active (Martin and Sloman, 2013). Based on our observations that brain activations associated with the similarity-based model was apparent in all conditions, while brain activations associated with the rule-based model was apparent only during the rule-based conditions, it is tempting to interpret our results in favor of a *default interventionism* account, where similarity-based processes act as default.

Our results might also be in line with the ideas of so called “*lazy algorithms*” (Aha, 1997) and with some early behavioral models. Early models of the conceptually similar task *function learning* (where one continuous cue is used to infer a continuous criterion, see e.g., Kalish et al., 2004) detailed the possibility that similarity-based processes underlie function learning but sometimes, in the case that a new function assessment scenario requires it, abstraction and extrapolation from the responses suggested by the learned exemplars can take place “on demand” (DeLosh et al., 1997). However, behavioral studies on judgment suggest that abstraction takes place already during learning (e.g., Juslin et al., 2008) whereby further research is needed to more clearly establish a relation between our results and the work on function learning.

The observation that similarity-based processes were engaged across all conditions, even in conditions where the overt response is guided by rule-based processes, might be used to understand demonstrations in behavioral studies that similarity-based considerations are hard to resist when making inferences (Hahn et al., 2010; von Helversen et al., 2014a; Bröder et al., 2017).

### Limitations and Future Studies

How do our results compare to neurocognitive accounts of “dual systems”? Arguably, the most influential account is the computational neuropsychological theory of category learning COVIS (COmpetition between Verbal and Implicit Systems; Ashby et al., 1998). In this theory it is suggested that category learning is governed by two neurally separable systems, one operating by verbalizable rules predominantly governed by prefrontal cortex, and the other via implicit, procedural processes primarily thought to involve the body and tail of the caudate. Recently, Ashby and Rosedahl (2017) suggest in a simulation study an interpretation of the *EBM* as part of the implicit system of the model COVIS. Future studies should be designed to specifically target the role of procedural processes in human judgment, for example by investigating to what extent the caudate can be related to an exemplar-based learning process.

Future studies should also be devoted to specifying the role of precuneus for human multiple-cue judgment in more detail, and aim toward a more mechanistic account than presented here. Such studies could continue to take advantage of relating psychological model-based measures to brain activation, potentially by using less coarse measures of cognitive processing than model fit. For example, experiments can be designed such that quantified processing components key for exemplar-based processing in judgment can be related to patterns of activation in the precuneus (see e.g., Nosofsky et al., 2012; Mack et al., 2013; Davis and Poldrack, 2014; Davis et al., 2017; Bowman and Zeithamova, 2018).

Our findings should be taken as a first attempt to use conjunction analysis of fMRI data in combination with behavioral analysis with cognitive modeling to investigate the relationship between associative and analytical judgments. Further research could take advantage of specific fMRI experimental protocols or brain imaging methods with a better temporal resolution than fMRI (e.g., EEG) to more in detail probe the question of the nature of the dynamic interplay between the two types of processes. For example, one possibility is that we would observe that precuneus activity precedes activity in superior frontal gyrus in all conditions, which could be taken as further support for our interpretation that similarity-based processes are routinely engaged for judgment rather than being engaged in an *either-or* fashion.

Previous brain imaging studies investigating brain activation differences between rule-based and similarity-based inferences have differed notably on the type of baseline task that is used for the contrast analyses (e.g., Patalano et al., 2001; Grossman et al., 2002; Koenig et al., 2005; von Helversen et al., 2014b; Milton et al., 2017). Our choice to use a rather strict comparison task is to increase the possibility that the activations resulting from the conjunction analyses can actually be said to be related to the judgment component of the task instead of other kinds of processes that are likely to be shared between conditions.

Finally, it should be noted that it is common to observe large individual differences in human judgment in general (e.g., Stanovich, 1999), so also in multiple-cue judgment (e.g., Hoffmann et al., 2014). We believe our study makes an important contribution to the field by linking individual differences in brain activation to adherence to specified cognitive models.

## Conclusion

Through investigating brain activity modulations shared between associative and analytic human judgments, and whether such activity relates to cognitive modeling of the processes, we suggest a similarity-based process for judgment, involving precuneus, that is routinely engaged both when responses are guided by similarity-based processes and rule-based processes. Analytical processes supported by regions such as superior frontal gyrus (BA6) presumably add on to the similarity-based considerations and together these mechanisms can produce adaptive judgment and decision making. Our findings thus support an *interplay* view on how these two types of processes interact, rather than a strict dichotomy, where the processes are executed in an *either-or* fashion.

## Author Contributions

LKW, SS, JE, PJ, and LN contributed to conception and design of the study, and wrote sections of the manuscript. LKW and SS were responsible for data collection. LKW, SS, and JE performed the statistical analyses. LKW wrote the first draft of the manuscript. All authors contributed to manuscript revision, read and approved the submitted version.

## Conflict of Interest Statement

The authors declare that the research was conducted in the absence of any commercial or financial relationships that could be construed as a potential conflict of interest.
