# Supplementary material for: A Similarity-Based Process for Human Judgment in the Parietal Cortex
Source: Front Hum Neurosci. 2018 Dec 13;12:481. doi: 10.3389/fnhum.2018.00481 (PMC6315133; doi:10.3389/fnhum.2018.00481)
Supplement: Supplementary file 1 [file Table_1.DOCX]

|  | CAM | EBM |
| --- | --- | --- |
| Condition | *ω*_1_, *ω*_2_, *ω*_3_, *ω*_4_, *ω*_5_, *k* | *s*_1_, *s*_2_, *s*_3_, *s*_4_, *s*_5_ |
| Instructed EBM | 4.36, 1.02, .89, -1.13, -.93, 14.81 | .08, .28, .30, .31, .35 |
| Instructed CAM | 5, 2.7, 2.6, 0.90, 1.15, 16.1 | .06, .12, .24, .63, .58 |
| Spontaneous EBM | 3.57, 3.81, 2.58, .82, -.41, 7.73 | .04, .06, .23, .33, .42 |
| Spontaneous CAM | 4.53, 3.75, 2.76, 1.61, .49, 14.95 | .04, .09, .14, .19, .68 |

Table S1

Mean best fitting parameter values for *CAM* and *EBM*, separately for the different conditions
