# Supplementary material for: A Similarity-Based Process for Human Judgment in the Parietal Cortex
Source: Front Hum Neurosci. 2018 Dec 13;12:481. doi: 10.3389/fnhum.2018.00481 (PMC6315133; doi:10.3389/fnhum.2018.00481)
Supplement: Supplementary file 2 [file Table_2.DOCX]

Table S2

Whole-brain correlation analysis with model fit of *EBM*

|  | | |  |  |  |  |  |  |  | |  |
| --- | --- | --- | --- | --- | --- | --- | --- | --- | --- | --- | --- |
|  |  |  |  |  |  |  |  |  |  | |  |
|  |  |  |  |  |  |  |  |  |  | |  |
|  | Cluster# | Region | BA | *x* | *y* | *z* | *t*-value | Voxels (*k*) |  | |  |
|  |  |  |  |  |  |  |  |  |  | |  |
|  | 1 | Medial superior frontal gyrus | 8 | -8 | 30 | 52 | 5.11 | 29860 |  | |  |
|  |  | White matter |  | -12 | 24 | 12 | 4.97 |  |  | |  |
|  |  | Medial superior frontal gyrus | 10 | -10 | 58 | -2 | 4.59 |  |  | |  |
|  |  | Hippocampus |  | -32 | -24 | -14 | 4.54 |  |  | |  |
|  |  | Hippocampus |  | 32 | -12 | -20 | 4.52 |  |  | |  |
|  |  | Precuneus | 31 | -8 | -60 | 30 | 4.42 |  |  | |  |
|  |  | Middle frontal gyrus | 10/46 | -38 | 48 | 14 | 4.29 |  |  | |  |
|  |  | Middle frontal gyrus | 10/46 | 34 | 50 | 18 | 4.16 |  |  | |  |
|  | 2 | Angular gyrus | 39 | -38 | -80 | 32 | 4.17 | 393 |  | |  |
|  | 3 | Cerebellum |  | 14 | -78 | -32 | 3.24 | 169 |  | |  |
|  | 4 | Middle frontal gyrus | 6 | -36 | 8 | 54 | 3.03 | 95 |  | |  |
|  | 5 | Inferior temporal gyrus | 37 | 50 | -68 | -10 | 3.01 | 166 |  | |  |
|  | 6 | Cerebellum |  | 12 | -48 | -42 | 3.00 | 110 |  | |  |
|  | 7 | Middle frontal gyrus | 9 | -48 | 18 | 40 | 2.92 | 35 |  | |  |
|  | 8 | Brain stem |  | -10 | -8 | -6 | 2.78 | 30 |  | |  |
|  | 9 | White matter |  | -16 | -78 | 6 | 2.57 | 29 |  | |  |
|  | 10 | White matter |  | -2 | 6 | -2 | 2.51 | 1 |  | |  |
|  | 11 | Superior frontal gyrus | 6 | 22 | 26 | 62 | 2.50 | 2 |  | |  |
|  | 12 | Superior occipital gyrus | 7 | -18 | -84 | 44 | 2.47 | 4 |  | |  |
|  | 13 | White matter |  | -48 | -36 | -14 | 2.46 | 4 |  | |  |
|  | 14 | Inferior frontal sulcus | 45/46 | 44 | 30 | 22 | 2.44 | 2 |  | |  |
|  | 15 | Superior frontal gyrus | 9 | 20 | 58 | 30 | 2.44 | 5 |  | |  |
|  | 16 | White matter |  | 26 | -30 | 50 | 2.42 | 4 |  | |  |
|  | 17 | White matter |  | 34 | -62 | 0 | 2.41 | 1 |  | |  |
|  | 18 | Middle frontal gyrus | 46 | 50 | 32 | 26 | 2.41 | 1 |  | |  |
|  | 19 | White matter |  | -52 | -4 | -24 | 2.41 | 2 |  | |  |
|  | 20 | White matter |  | 12 | 56 | 10 | 2.40 | 1 |  | |  |
| *Note*. BA = Brodmann area. Coordinates (x, y, z) in MNI space (SPM8). *t*-values at the peak voxel. Voxel: *p* < .05 (FDR-corrected) Cluster: *k* > 0. | | | | | | | | | |  | |
