# Supplementary material for: A Similarity-Based Process for Human Judgment in the Parietal Cortex
Source: Front Hum Neurosci. 2018 Dec 13;12:481. doi: 10.3389/fnhum.2018.00481 (PMC6315133; doi:10.3389/fnhum.2018.00481)
Supplement: Supplementary file 3 [file Table_3.docx]

Table S3

Whole-brain correlation analysis with model fit of *CAM*

|  | | |  |  |  |  |  |  |  |
| --- | --- | --- | --- | --- | --- | --- | --- | --- | --- |
|  |  |  |  |  |  |  |  |  |  |
|  |  |  |  |  |  |  |  |  |  |
|  | Cluster # | Region | BA | *x* | *y* | *z* | *t*-value | Voxels (*k*) |  |
|  |  |  |  |  |  |  |  |  |  |
|  | 1 | Parieto-occipital sulcus | 7/19 | -16 | -82 | 48 | 5.83 | 57366 |  |
|  |  | White matter |  | -34 | -64 | 4 | 5.70 |  |  |
|  |  | Central sulcus | 4 | 62 | -2 | 22 | 5.33 |  |  |
|  |  | Middle frontal gyrus | 8 | -40 | 28 | 38 | 5.19 |  |  |
|  |  | Supramarginal gyrus | 40 | -52 | -42 | 52 | 4.66 |  |  |
|  |  | Precentral sulcus | 6 | -42 | -4 | 60 | 4.62 |  |  |
|  |  | Medial superior frontal gyrus | 6 | -4 | 2 | 64 | 4.60 |  |  |
|  |  | Inferior temporal gyrus | 37 | -52 | -66 | -8 | 4.43 |  |  |
|  |  | Superior parietal lobe | 7 | 36 | -46 | 68 | 4.41 |  |  |
|  |  | Superior frontal gyrus | 4 | -24 | 4 | 68 | 4.39 |  |  |
|  |  | Superior temporal sulcus | 22 | -50 | -54 | 14 | 4.31 |  |  |
|  |  | Medial superior frontal gyrus | 6 | 2 | -2 | 72 | 4.08 |  |  |
|  | 2 | Inferior occipital gyrus | 17/18 | -14 | -100 | -12 | 3.58 | 53 |  |
|  | 3 | Superior temporal sulcus | 21 | 52 | 6 | -18 | 2.89 | 70 |  |
|  | 4 | Inferior occipital gyrus | 18 | -24 | -80 | -20 | 2.32 | 13 |  |
|  | 5 | White matter |  | 10 | 42 | 44 | 2.30 | 5 |  |
|  | 6 | Inferior occipital gyrus | 18 | 28 | -94 | -10 | 2.25 | 2 |  |
|  | 7 | White matter |  | 20 | -46 | 26 | 2.24 | 9 |  |
|  | 8 | Medial superior frontal gyrus | 8 | 2 | 56 | 40 | 2.23 | 1 |  |
|  | 9 | Putamen |  | -20 | 2 | -8 | 2.20 | 1 |  |
|  | 10 | Middle occipital gyrus | 18 | 18 | -98 | -2 | 2.18 | 1 |  |
| *Note*. BA = Brodmann area. Coordinates (x, y, z) in MNI space (SPM8). *t*-values at the peak voxel. Voxel: *p* < .05 (FDR-corrected) Cluster: *k* > 0. | | | | | | | | |  |
